# Gonadotropin Cell Transduction Mechanisms

**DOI:** 10.3390/ijms23116303

**Published:** 2022-06-04

**Authors:** Annunziata Mauro, Paolo Berardinelli, Barbara Barboni

**Affiliations:** Faculty of Bioscience and Technology for Food, Agriculture and Environment, University Teramo, Via R. Balzarini 1, 64100 Teramo, Italy; pberardinelli@unite.it (P.B.); bbarboni@unite.it (B.B.)

The intention of this Special Edition was to collect review and original research articles that illustrate and stimulate the growing efforts to highlight the mechanisms of action of gonadotropins, as well as deepen our understanding of their biological roles in health and disease, aiming at revealing novel therapeutic opportunities in reproductive and regenerative medicine. Gonadotropins act in concert in the regulation of the female and male reproductive systems. Through intricate endocrine communication routes, and through paracrine and autocrine communication along the HPG axis, they sustain the production of sex steroids by gonads. Different signaling cascades and transcriptional mechanisms are activated according to the variation in GnRH pulse frequency, after the synthesis and release of FSH and LH, whose biological effects are mediated directly by coupled receptors (FSHR and LHCGR) or indirectly through different mediators. The orchestrated signaling activation promotes different biological responses, such as ovarian angiogenesis, folliculogenesis, oocyte selection, fertilization, and pregnancy, as well as the testicular development and spermatogenesis. Despite multiple signaling cascades being simultaneously activated in the target cells [1], a limited number of these pathways were known for decades, but an increasing body of evidence emerging from the recent literature is uncovering a more complex and complete view of the action of these hormones. Many efforts have been made to clarify in particular the mechanisms related to FSH/FSHR system as evidenced in the papers by De Pascali et al. [2] and Recchia et al. [3]. The FSHR belongs to the family of G protein-coupled receptors (GPCRs), playing a key role in the activation of Gαs/PKA/cAMP, PKC/MAPK, and Ca+/CaMKII pathways [2,3]. However, FSHR can also be found in extra-gonadal organs and tumorous tissues, such as different types of cancer, tumor vessels, endothelial cells, osteoclasts, human umbilical vein endothelial cells, monocytes, the liver, and in a population of stem cells called very small embryonic-like stem cells (VSELs), as thoroughly reported in the review by Recchia et al. [3]. Most of all, in this overview, in particular, the most relevant research on the mechanisms through which FSH is involved in germ-cell development cells in vivo and in vitro during reproductive life have been discussed. The authors described the influence of FSH on spermatogenesis and folliculogenesis, mainly in the germ cells of humans and other species, underling the molecules and signaling pathways implicated in each phase of the processes [3]. Through FSHR in the membrane of Sertoli cells, FSH binds to these cells and stimulates the release of factors that help in self-renewal, such as GDNF and FGF2, and the differentiation (BMP4, activin A, and KL) of spermatogonial stem cells (SSCs). Therefore, FSH deficiency in mice and humans can reduce spermatogenesis and azoospermia in some cases. It was also shown that the addition of FSH with other factors (such as testosterone) could promote the differentiation of PGCLCs into mature gametes in vitro [3].

The FSH/FSHR system dysregulations are involved in several reproductive disorders in women (such as premature ovarian insufficiency, polycystic ovarian syndrome, ovarian hyperstimulation syndrome) and in men (such as male idiopathic infertility). However, the plethora of activated signaling pathways makes it difficult to determine which signaling module is involved in each pathology. Many studies have made it important to have molecules capable of biasing signaling highlighting at the FSHR level; this point of emphasis is an important pharmacological tool to understand the specific FSHR signaling pathway contributing to pathologies, as well as to develop a new generation drugs devoid of the adverse side effects of current reproductive clinical treatments. In this context, De Pascali et al. [2] demonstrated that the low molecular weight (LMW) ligands endowed with biased agonist properties were particularly helpful tools for deciphering complicated FSH/FSHR signaling systems because they allowed selective activation of distinct signaling cascades. Indeed, in comparison to FSH, three benzamide and two thiazolidinone LMW derivatives were studied in depth for their pharmacological variety in wildtype, Gαs, or β-arrestin 1/2 CRISPR knockout HEK293 cells. The results showed that each LMW ligand presented a discrete pharmacological efficacy compared to FSH. Interestingly, these LMW ligands were able to generate kinetic profiles distinct from FSH (i.e., faster, slower or transient, depending on the ligand) correlated with CRE-dependent transcription. The possibility of using LMW as pharmacological tools for dissecting and assessing the relative contributions of signaling pathways activated by FSHR at the cellular and physio-pathological levels [2] is particularly useful in improving the assisted reproductive techniques (ART) in which ovulation is induced through the injection of FSH preparations in controlled ovarian stimulation, a procedure that has FSHR as a target.

In the review by Lee et al. [4] a directed focus on Estrogen receptors (ERs), and in particular the role of estrogen receptor type β (ERβ) regulating gonadotropin response during folliculogenesis, were disserted. Gonadotropin receptors are expressed in TCs and GCs. The presence of ERα in theca (TCs), and ERβ in granulosa (GCs) cells of ovarian follicle are essential for gonadotropin-induced steroidogenesis and gametogenesis. Even though it remains unclear how gonadotropin signaling interacts with estrogen signaling and the hierarchy in these signaling mechanisms in those somatic cells, it has been reported that FSH-induced *Lhcgr* expression in GCs depends on the presence of ERβ [5]. Given that they observed the loss of ERβ reduced estrogen synthesis in GCs, the authors hypothesized that ERβ-dependent estrogen signaling can positively regulate *Lhcgr* gene expression in GCs. In contrast, since the expression of *Fshr* was increased in the absence of ERβ in the ovary [5], it has been suggested that ERβ may negatively regulate *Fshr* expression in GCs. Despite the fact that molecular mechanisms underlying ERβ regulation of gonadotropin receptors in GCs remain unknown, Lee et al. [4] observed that a subset of gonadotropin-induced genes in GCs, which are essential for ovarian follicle development, oocyte maturation, and ovulation, are dependent on ERβ.

In the study by Di Berardino et al. [6], Equine Chorionic Gonadotropin (eCG) was proposed as an alternative to FSH in the in vitro folliculogenesis (*iv*F) system, representing a valid and largely available hormonal support enabling a synchronized in vitro follicle and oocyte development. The ability to reproduce in vitro the first phases of folliculogenesis (follicle growth and oocyte development) still represents an unsolved challenge for reproductive biotechnology in mammals species, as it requires wide margins of standardization, such as the definition of the gonadotropic role [7]. Di Berardino and co-workers were able to compare and validate the hormonal effects of 3D *iv*F on single-ovine preantral (PA) follicles within two different cultural contexts. By enhancing its influence under FBS-free media, eCG assisted to stimulate the in vitro development of ovine PA follicles. Follicular growth, antrum formation, steroidogenic activity, and gap-junction marker expression all showed improvements. Furthermore, it has been demonstrated that eCG had a beneficial effect on the germinal compartment, resulting in a higher rate of meiotic competent oocytes [6]. Given that *iv*F could be a feasible way for delivering large number oocytes in ART, since various attempts have made to establish *iv*F culture procedures beginning with the selection of the optimal gonadotropic stimulus, these findings should help to broaden the use of eCG to *iv*F as a widely used hormonal support permitting a valid synchronized in vitro reproduction.

In another work, Hsu et al. [8] investigated the effect of recombinant human FSH (rhFSH) on ovarian follicle and uterine parameters after abdominal and vaginal in vivo administration in infertile women receiving clinical in vitro fertilization (IVF). Detection and retrieval of mature oocytes from a human ovary is the first mandatory pre-requisite and controlled ovarian stimulation (COS) involving gonadotropin administration to stimulate the growth of multiple ovarian follicles, and it is the most effective way to increase pregnancy rates in IVF treatments. Furthermore, the authors compared the pharmacokinetic parameters from serum and urine samples in non-infertile women who have received a stimulation of 300 IU rhFSH between abdominal and vaginal administrations. At the same time, the effects of injected rhFSH on the uterine endometrium, ovarian follicle growth, and functional parameters (ER and PR) have also been investigated in female rats. All the results of this study suggest various scenarios for controlled ovarian stimulation (COS) in clinical IVF treatment.

In the female reproductive system, gonadotropins play a key role in the development of an adequate blood vessel network necessary to support the proliferative and endocrine functions of the follicular cells and crucial for the accomplishment of ovarian follicle growth and ovulation. Vascular Endothelial Growth Factor (VEGF) through gonadotropins guides ovarian angiogenesis. In particular, in the research article by Mauro et al. [9], it shows that Luteinizing hormone (LH) surge, during a short window lasting from 24 to 44 h depending on the species, is responsible for triggering the profound morphological and functional changes occurring during the transition from a preovulatory follicle to a periovulatory before it ovulates [9]. The study aimed to clarify in a pig model the role of P_4_ in controlling ovarian angiogenesis with a spatio-temporal VEGF-mediated mechanism during the periovulatory phase. The results demonstrated that VEGF receptors (FLT1 and FLK1) and the related downstream ERKs and AKTs pathways were affected by the in vivo administration of the P4 antagonist, RU486, during the transition from the pre to periovulatory phase. In addition, it has been demonstrated that RU486 was able to inhibit specifically the VEGF-dependent angiogenetic mechanisms during the periovulatory phase in a compartment- and time-dependent manner. Indeed, MAPK/ERKs pathway widely prevailed in the GR compartment of preovulatory follicles, whereas PI3K/AKTs were operative in the TC layer. The hCG treatment, by mimicking the LH surge, strongly activated both pathways. Most interestingly, both the downstream kinases of VEGF pathway could be antagonized by blocking the P4 action, and this process was associated with profound morphological and functional follicular changes during the periovulatory phase. It have demonstrated that RU486 administration during the periovulatory phase inhibit the VEGF signaling pathways mainly in the TC compartment by contributing to a downregulation of GR and TC ERKs and AKTs activations [9]. These evidences provide new insights into the biological in vivo role of the P4 in driving vascular and tissue remodeling during the transition from the preovulatory to the periovulatory stage by introducing a new role in controlling female reproductive outcomes.

In the review by Shim et al. [10], it is reported the recent advances in the study of genes mutation related to the Notch signaling pathway that was found in patients with central precocious puberty (CPP). CPP is characterized by the early activation of the hypothalamus-pituitary-gonadal (HPG) axis. CCP people show progressive sexual development and testicular enlargement before 8–9 years of age, and they have elevated gonadotropins levels. Idiopathic CPP, defined as CPP with no specific organic cause, is found in 90% to 95% of girls and 50% of boys with CPP, respectively. Actually, CPP is treated with a GnRH agonist, which has a longer and stronger action time compared with that of endogenous GnRH. It downregulates GnRH receptor expression and suppresses gonadotropin secretion. Although the mechanism underlying CPP is not clear, it is thought that it involves a complex interplay of genetic and environmental factors. In particular, it has been reported that genetic factors influence CPP development in 50–80% of CPP cases. Although genetic factors play an important role in CPP development, few associated genetic variants have been identified. Experimental evidences indicated that aberrant Notch signaling may be associated with abnormal pubertal development. However, the available research evidence so far remains inconclusive, and more studies will be necessary to confirm or refute the suggested role of the Notch signaling pathway in pubertal development.

The complex interplay of gonadotropins signal transduction pathways in the human reproductive system is one of the most topical biomedical subjects of inquiry, and it is earnestly hoped that the contributions to this Special Issue will provide some insight and help in the ongoing efforts to better clarify their mechanisms’ actions and reveal novel therapeutic opportunities for reproductive diseases.
